# Properties of Paramagnetic Metals in MRI

**DOI:** 10.1155/MBD.1997.159

**Published:** 1997

**Authors:** Michel Schaefer

**Affiliations:** Laboratoire GUERBET, 18-24 rue Jean Chaptal Aulnay-sous-Bois B.P. 50400 Roissy CdG Cedex F-95943 France

## Abstract

The use of different metals in Magnetic Resonance Imaging will be briefly described.

This overview will be more a qualitative description than a full paper on this topic, in other words, this paper contributes to explaining how and why some metal ions have been chosen for MRI.

Paramagnetic compounds are currently used in clinical practice. The main differencesin the four gadolinium complexes come from their different chemical structures. The macrocyclic compounds display a high symmetry and rigidity and therefore a high level of stability.

All these four complexes display the same efficacyon the signal in MRI. In addition, they are very well tolerated.

The second main class of compounds consists of the iron oxide superparamagnetic particles. They are used in the detection of focal liver lesions. Through their action in dephasing the spin, they are used as negative enhancers.


### PROPERTIES OF PARAMAGNETIC METALS IN MRI

Michel Schaefer

Laboratoire GUERBET, 18-24, rue Jean Chaptal,
Aulnay-sous-Bois - B.P. 50400, F-95943 Roissy CdG Cedex, France

**ABSTRACT :**
The use of different metals in Magnetic Resonance Imaging will be briefly described.
This overview will be more a qualitative description than a full paper on this topic, in other words, this paper contributes to explaining how and why some metal ions have been chosen for MRI.
Paramagnetic compounds are currently used in clinical practice. The main differencesin the four gadolinium complexes come from their different chemical structures. The macrocyclic compounds display a high symmetry and rigidity and therefore a high level of stability.
All these four complexes display the same efficacyon the signal in MRI. In addition, they are very well tolerated.
The second main class of compounds consists of the iron oxide superparamagnetic particles. They are used in the detection of focal liver lesions. Through their action in dephasing the spin, they are used as negative enhancers.

### I. INTRODUCTION

Before entering into the details of Magnetic Resonance Imaging, it is worth comparing two of the most important tools used in medical diagnostics : X-rays and MR imaging.

These two modalities can be compared in terms of differences and complementarities (fig 1).
X-Ray imaging needs high energy and radiations. Essentially hard tissues like bones can be visualised. In fact, the heavy metals like calcium interact with X-Rays and give off a signal. This signal is obtained through the interaction between the X-Ray beam and the electronic shell of atoms with high atomic numbers.

Thus, the best contrast media for X-Ray imaging will be molecules or atoms with high atomic numbers and a k-Edge in the range of X-Ray energy used in scanner machines. Direct interaction is obtained between iodinated molecules which are used as contrast media in this technique and X-Rays. This explains why the technique requires large amount of contrast media. In other words, the contrast media are directly « seen » on the image.

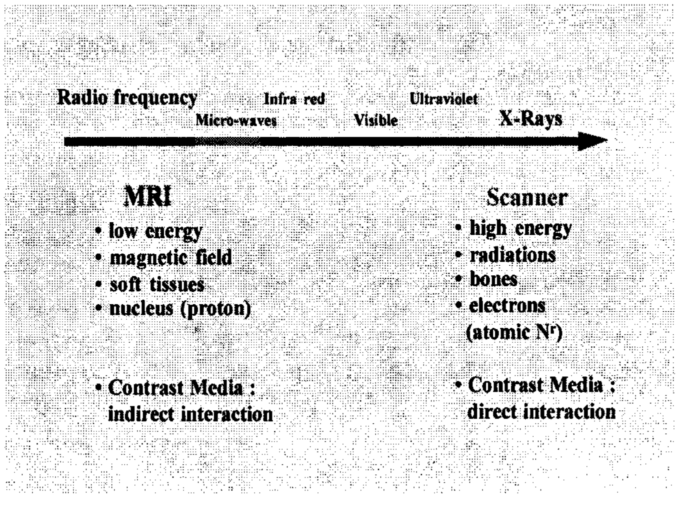

*Fig.1: Differences and complementarity of X-ray and MR imaging techniques. From : An Introduction To MR in Medicine (Ref. 1).*

Conversely, magnetic resonance imaging does not require high energy. The patient is introduced into a magnetic field. In MRI, it is the soft tissues which are visualised best. The images are obtained with the signal coming from the lipid and water protons. The image contrast is obtained through the relaxation times T1 and T2 of the different tissues. Injection of contrast media will modify both relaxation times T1 and T2 and then induce contrast enhancement. There is an indirect interaction between the contrast media and the signal. This explains the relatively low amount of substance injected in comparison with X-Ray modalities. Unlike the results obtained with X-rays, the contrast material is not directly visualised in MRI.

### II - CONTRAST IN MAGNETIC RESONANCE IMAGING

The contrast between the tissues in MRI mainly comes from three parameters : the amount of protons, so called proton density, and their two relaxations times T1 and T2.

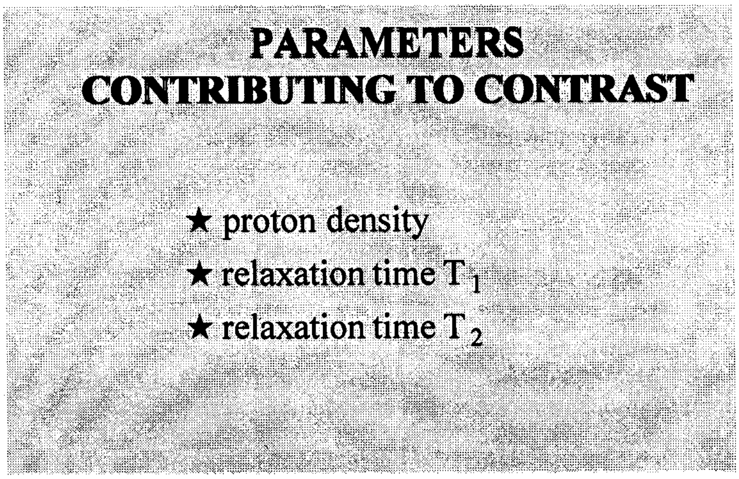

***Fig 2 : the three main parameters on which we can act to modify the contrast.***

As it is difficult and dangerous to modify the amount of water or fat in vivo, the substances used to increase the contrast will act in modifying the relaxation times (fig.2). It can be demonstrated that the dipolar interaction between free or unpaired electrons and water molecules induces a dramatic decrease in T1 and T2.

### III. PARAMAGNETIC COMPOUNDS :

| ELEMENT | CONFIGURATION | | | | | | | Nb of SPIN |
|---|---|---|---|---|---|---|---|---|
| $Ti^{2+}$ | ↑ | ↑ | – | – | – | | | 2/2 |
| $Cr^{3+}$ | ↑ | ↑ | ↑ | – | – | | | 3/2 |
| $Mn^{2+}$ | ↑ | ↑ | ↑ | ↑ | ↑ | | | 5/2 |
| $Fe^{3+}$ | ↑ | ↑ | ↑ | ↑ | ↑ | | | 5/2 |
| $Fe^{2+}$ | ↑↓ | ↑ | ↑ | ↑ | ↑ | | | 4/2 |
| $Co^{2+}$ | ↑↓ | ↑↓ | ↑ | ↑ | ↑ | | | 3/2 |
| $Ni^{2+}$ | ↑↓ | ↑↓ | ↑↓ | ↑ | ↑ | | | 2/2 |
| $Cu^{2+}$ | ↑↓ | ↑↓ | ↑↓ | ↑↓ | ↑ | | | 1/2 |
| $Gd^{3+}$ | ↑ | ↑ | ↑ | ↑ | ↑ | ↑ | ↑ | 7/2 |

PARAMAGNETIC IONS

***Table 1 : among the metals possessing unpaired electrons, Gadolinium, Manganese and Iron appear the most powerful with 7, 5 and 5 unpaired electrons, respectively.***

The best carriers of free -or unpaired- electrons, beside the free radicals are metals. Table 1 shows that the best metal ions are manganese and particularly gadolinium. Iron and gadolinium-based compounds are currently used in clinical practice. Manganese compounds are under clinical trial (Fig.3 summarizes the paramagnetic action).

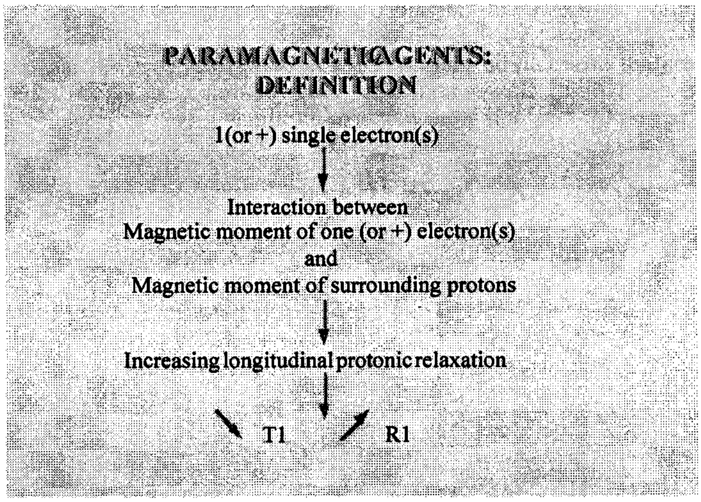

***Fig 3: Paramagnetic compounds are mainly used as positive enhancers in T1-weighted sequences.***

a - Mechanism:

The dipolar interaction between water molecule protons and unpaired electrons is described by the Solomon-Bloembergen equation (fig. 5).

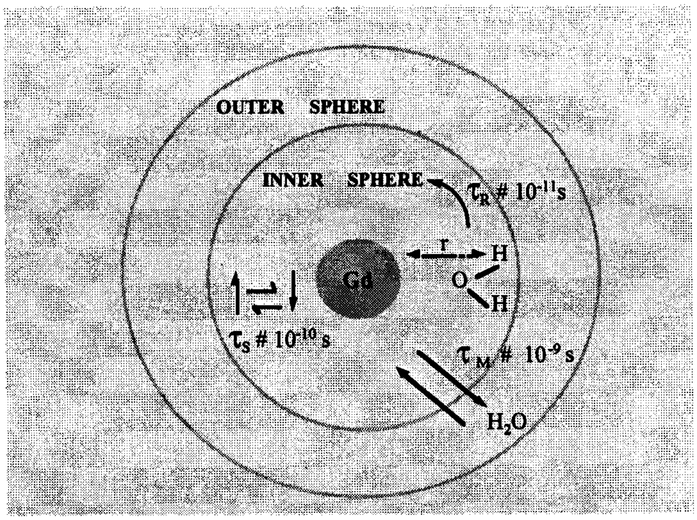

***Fig 4: The dipolar interaction between water molecules and metal ion is responsible for the paramagnetic efficacy. Two simultaneous phenomena contribute to the relaxivity: the water molecules from the inner sphere (directly bound to the metal) and free water molecules coming from the surrounding tissues.***

In these equations, two parameters are very important: r which is the distance between the metal and the proton of the water molecule. This distance must be as short as possible in order to increase the efficacy.If r increases slightly, $r^6$ increases dramatically and the efficacy almost vanishes (fig. 4).

The second important parameter is $\tau c$ which represents the movements of the paramagnetic molecule. This correlation time is composed of three parameters :

- $\tau_s$ which is the electronic correlation time related to the symmetry and rigidity of the gadolinium complexes. From this point of view, the macrocyclic complexes are advantageous as they display a higher symmetry than the linear complexes and therefore have a longer electronic correlation time. This is very important when synthesizing new compounds with increased relaxivity. As the correlation time must be as high as possible, it is understandable that this electronic correlation time should not be a limiting factor (fig. 6).
- $\tau_R$ the rotation correlation time can be increased synthesizing bigger molecules. In this way, the rotation rate of such big molecules will be slower and thus their rotation correlation time longer. This is a very important point in the search for new and more powerful products.

SOLOMON-BLOEMBERGEN EQUATION

$$\frac{1}{T_{1M}} = \frac{2}{15} \cdot \frac{S(S+1)\gamma^2 g^2 \beta^2}{r^6} \left[ \frac{3\tau_c}{1+\omega_i^2 \tau_c^2} + \frac{7\tau_c}{1+\omega_s^2 \tau_c^2} \right]$$

$$\frac{1}{\tau_c} = \frac{1}{\tau_R} + \frac{1}{\tau_s} + \frac{1}{\tau_M}$$

***Fig 5: Solomon/Bloembergen equation. The paramagnetic efficacy, the relaxivity, depends chiefly on three parameters : the number of water molecules around the metal, the distance r between them and the correlation time $\tau c$.***

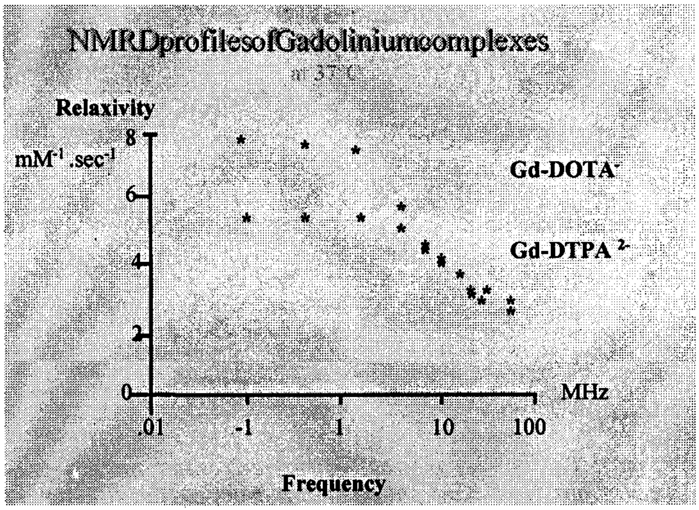

***Fig 6: At low field strengh, the higher relaxivity displayed by the macrocycle is due to its longer electronic correlation time $\tau s$ because of the higher symmetry and rigidity of the macrocyclic structure.***

- $\tau_M$ the exchange correlation time is the most difficult to manage as it appears in two equations (fig. 7). This correlation time represents the residence time for a water molecule fixed to the metal ion. It must represent the best compromise between a long period of time in order for a water molecule to be relaxed, and a short residence time to ensure the fast turn over of other molecules. In other words, $\tau_M$ should not be a limiting factor. The precise analysis of all these correlation times is perfectly described in Merbach's paper (ref. 2).

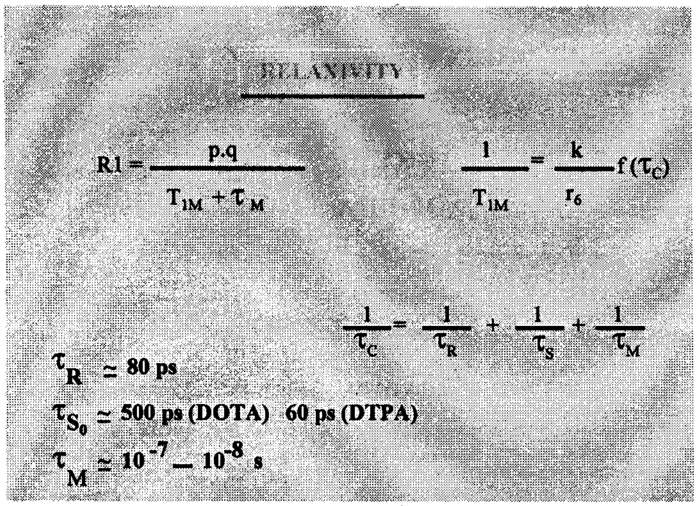

***Fig 7: The exchange correlation time appears in the two equations and need to be well managed. In other words, the best compromise must be found concerning its value.***

The gadolinium metal ion with nine water molecules in its first hydration sphere is very efficient with a relaxivity of 9 mM-1. s-1, but it is toxic in vivo (fig. 8). In fact, the gadolinium with an ionic radius similar to the calcium ion, can interfere with numerous metal-dependent enzymatic systems. Therefore, it must be complexed in order to mask its toxicity, but this results in decreased relaxivity (table 2).

b - Tolerance:

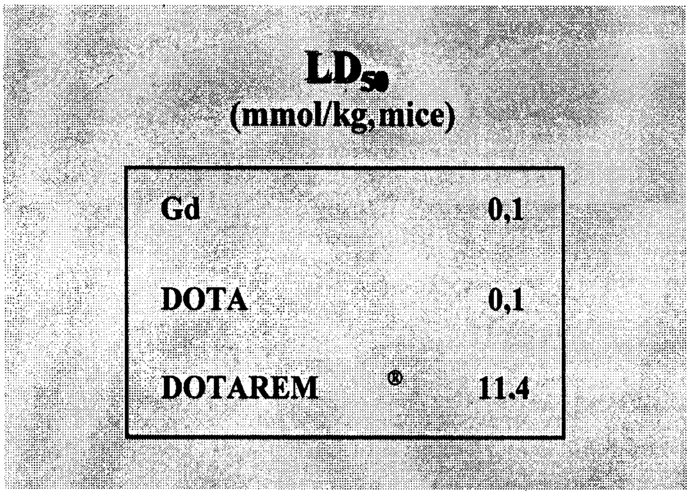

***Fig 8: Gadolinium metal ion is toxic through its possible interference with metal dependent systems. The plain chelate is also toxic as it can complex any endogenous metal. The gadolinium decomplexation by the appropriate chelate allows a gain in tolerance by a factor more than 100.***

| T1 at 90 MHz (msec) (0.001 M solutions) | |
|---|---|
| $MnCl_2$ | 400 |
| Mn-DTPA | 698 |
| FeCl3 | 340 |
| Fe-DTPA | 886 |
| $GdCl_3$ | 81 |
| Gd-DTPA | 137 |

***Table 2: Whatever the metal, complexation leads to a decrease in relaxivity. By surrounding the metals by a chelate, the water molecules are removed. Free gadolinium ion possesses 9 water molecules and just 1 when complexed.***

Four gadolinium complexes are currently available on the market. Most of their properties come from their different chemical structures (fig. 9). They can be divided in four main categories according to their structures: macrocyclic or linear, and according to their nature: ionic or non-ionic.

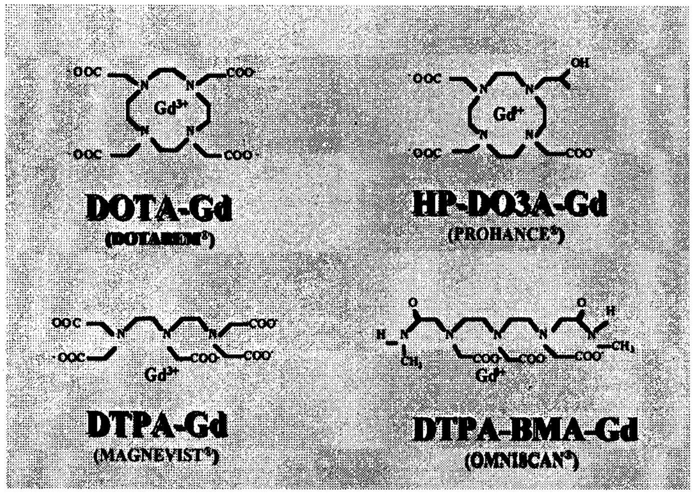

***Fig 9: Four gadolinium complexes are available on the International market. They differ by their chemical structures. Two main classes : linear and macrocyclic structures.***

Thanks to the low amount of compounds injected in MRI, the ionicity of the complexes is not a crucial factor as the increase of plasma osmolality is very low, unlike what is observed in X-Ray imaging. The stability of the complexes must be as high as possible to avoid any in-vivo decomplexation.

Macrocyclic complexes display the highest thermodynamic stability constant (fig. 10).

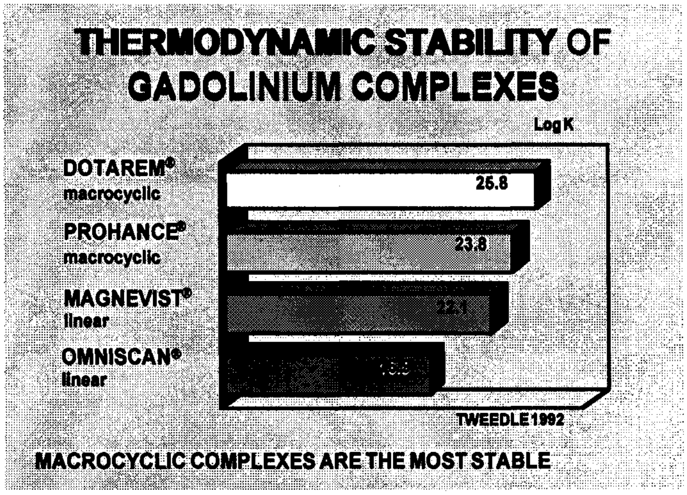

***Fig. 10: Thermodynamic constants show that macrocyclic structures are the most stable.***

Moreover, at physiological pH, the ionic complexes are the most stable (fig. 11).
Kinetic stability is also a very important factor as it conditions possible in-vivo transmetallations (table 3). In vitro, measurements confirm this behaviour when the four gadolinium complexes currently available on the market are placed in competitive conditions with zinc salt for example.

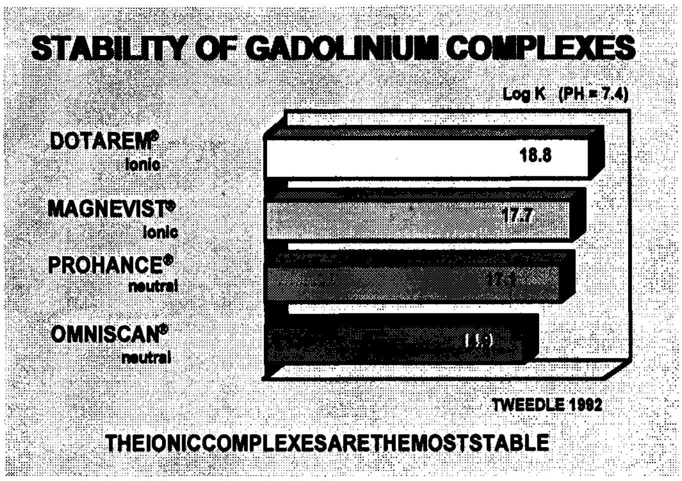

***Fig. 11: Conditional stability constants at physiological pH show that ionic structures are the most stable.***

Practically no exchange takes place with macrocycles (fig. 12).
In the same way, in-vivo gadolinium retention has been monitored in mice. After 14 days, the gadolinium in bones was below the limit of detection of the methods for the two macrocyclic complexes.

**EQUILIBRIUM CONSTANTS FOR GADOLINIUMCHELATES**

| | T1/2 dissociated 0.1 M HCl |
|---|---|
| Gd-HP-DO3A | 3 hrs |
| Gd-DOTA⁻ | > 1 mo |
| GD-DTPA-BMA | - 30 s |
| $Gd\text{-}DTPA^{2-}$ | 10 mins |

TWEEDLE MF. Invest. Radiol. 1992, 27, 52-56

***Table 3: This table displays the half-life of complexes placed in very acidic conditions in order to accelerate the decomplexation phenomenon and to allow its measurement.***

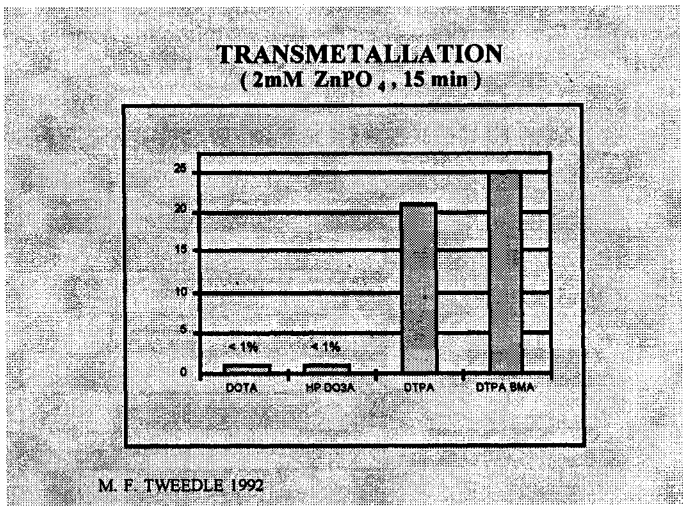

***Fig 12: This diagram shows the percentage of the exchange with zinc salt after _ hour in-vitro (ref. 3).***

The injected doses were 4 times the human clinical doses and the amounts found in bones were very low (fig. 13). We can thus conclude that all these 4 gadolinium complexes used as contrast media in MRI are very safe and well tolerated. The usual dose injected in humans is 0.1 mmol/Kg. These compounds are called non-specific as they are very hydrophilic, they do not bind to proteins or receptors, they are excreted unmetabolized in urine and are considered as extra-cellular fluid markers.

Some others metal complexes are under clinical trial as specific compounds. For example, lipophilic gadolinium complexes and Manganese complexes are studied as hepatocyte-targeted compounds for liver imaging (table 4).

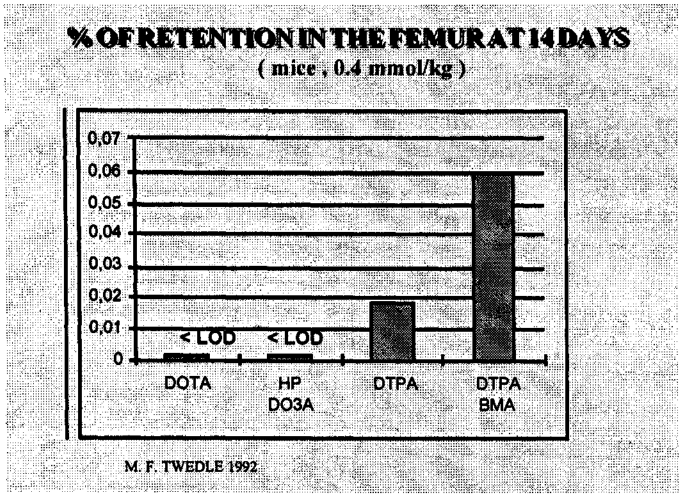

***Fig 13: percentage of Gd retention in bone in mice after 14 days (ref. 4).***

### IV. SUPER PARAMAGNETIC PARTICLES

Iron oxide particles represent the second most important class of metal derivatives used for MR imaging. These superparamagnetic particles are synthesized through a co-precipitation of FeII and FeIII in presence of dextran. Dextran is a polysaccharide used as a coating in order to stabilise the colloidal suspension (fig 14).

| | |
|---|---|
| **EXTRACELLULAR COMPOUNDS**<br>Gd-DOTA<br>Gd-DTPA<br>Gd-HPDO3A<br>Gd-DTPA BMA | **HEPATOBILIARY CONTRAST AGENTS**<br>Mn-DpDp<br>Gd-BOPTA<br>Gd-EOB DTPA |
| **MMPS-TARGETED AGENTS**<br>SPIO<br>ENDOREM®<br>RESOVIST® | **BLOOD-POOL**<br>MACROMOLECULES<br>USPIO (SINEREM®) |

***Table 4: Besides the four gadolinium complexes used as non-specific agents, other more specific compounds are under development. Among the liver-specific contrast agents, one superparamagnetic particle is on the market (ENDOREM® ).***

These iron oxide particles are called superparamagnetic as they have a high susceptibility as Ferro-magnetic compounds but they demagnetise very rapidly as paramagnetic molecules when the magnetic field is removed (fig. 15).

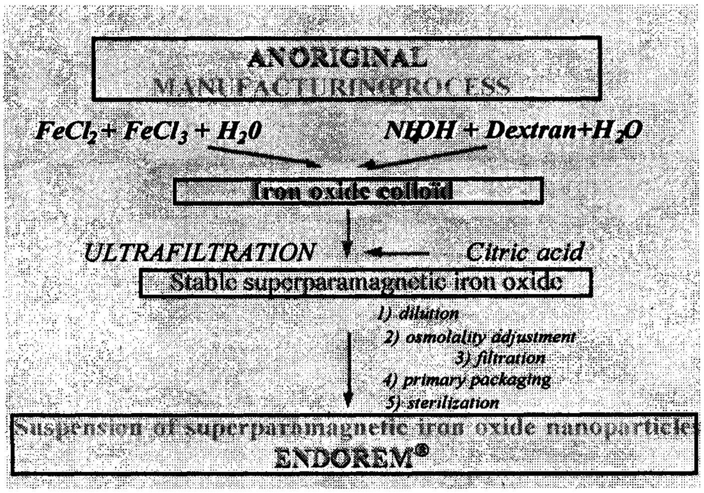

***Fig 14: Manufacturing process of superparamagnetic particles. After coprecipitation, the suspension is filtered and stabilized.***

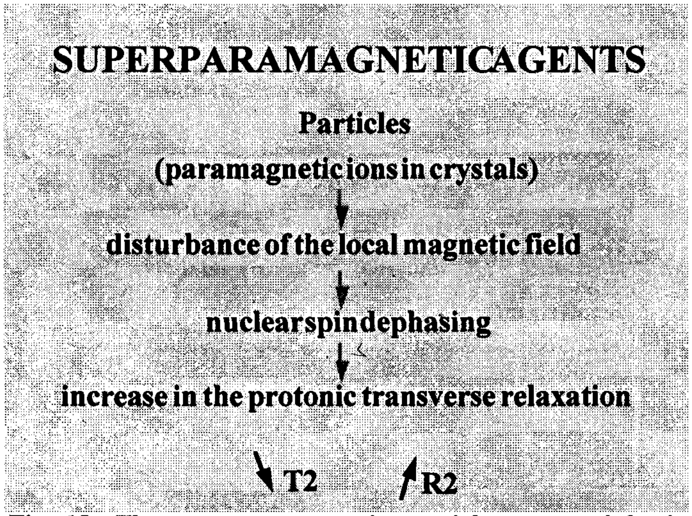

***Fig 15: The superparamagnetic particles act mainly in dephasing the spins. They are generally used as negative enhancers.***

The precise characterization of such particles is very important because their physico-chemical and pharmacokinetic properties will dramatically depend on their characteristics. Determination of the size of the iron oxide crystal itself is measured by sophisticated methods. This is important because their size will determine their magnetic properties (fig. 16).

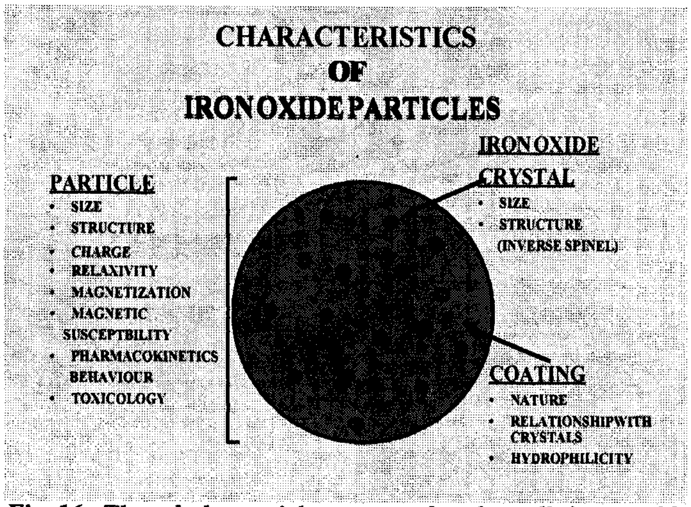

***Fig 16: The whole particles are made of small iron oxide crystals coated with dextran. Mannitol and citrate ensure an iso osmolality to the plasma and contribute to stability.***

**SUPERPARAMAGNETICPARTICLES**

| PRODUCTS | CRYSTAL SIZE nm | DIAMETEROF THEPARTICLE nm |
|---|---|---|
| LUMIREM® | 8,5 | 300 |
| ENDOREM® | 4,5 | 150 |
| SINEREM® | 4,5 | 30 |

***Table 5: Sizes of the particles.***
***The size of the iron oxide crystals is very similar whatever the compound. On the other hand, the size of the particle itself is absolutely determining as it will affect all the properties of the compound.***

The size of the particle is crucial (table 5). Particle size measurement is performed by laser light scattering. The largest particles are found in Lumirem which is an oral compound used to darken the bowels in order to better visualise the adjacent structures in abdominal and pelvic imaging (fig. 17).

**LUMIREM®**
**Ferumoxsil**

Indications:

. Negative marker of the GI tract in MRI

. Better delineation of adjacent structures.

***Fig 17: Lumirem is taken orally and helps to improve definition of the adjacent structures in the pelvis and abdomen.***

Its relaxivities in T1 and T2 show that this compound is nearly exclusively a T2 negative enhancer (table 6).

With a diameter of 150 nm, Endorem is a specific compound for liver imaging. The Küppfer cells present in normal parenchyma will take up the particles and then induce a T2 effect which will darken the normal liver. The malignant lesions which lack macrophages will not take up the particles and will appear in iso-signal. With a smaller diameter of around thirty nm, Sinerem will stay in the blood vessels for a longer period of time and can be used as a blood-pool agent. At delayed imaging, 24 h after injection, the lymph nodes can be visualised.

**SUPERPARAMAGNETIC PRODUCTS:**

**$r_1$ & $r_2$ RELAXIVITIES**

**Relaxivity ($mM^{-1}.s^{-1}$)**

| | r1 | r2 | r2/r1 |
|---|---|---|---|
| LUMIREM® | 3 | 74 | 25 |
| ENDOREM® | 25 | 100 | 4 |
| SINEREM® | 22 | 53 | 2.4 |
| GdChelates | 4.5 | 5.7 | 1.3 |

***Table 6: The relaxivity depends on the size of the particles. The T2/T1 effects ratio increases with the size. The larger the particles, the higher the T2 effect and the darker the image.***

Table 6 shows that relaxivities R1 and R2 and in particular R2/R1 depend mainly on their size. This table shows that these particles can be used as T1 and T2 agents. The enhancement in T1 will be obtained when the particles are diluted in the blood vessels for example, giving a so-called angio-effect. The negative enhancement in T2 is obtained when the particles are concentrated in the macrophages for example, inducing an important susceptibility effect.

### V. CONCLUSION

In order to use all the compounds under optimal conditions, their precise mechanism should be understood perfectly. With a constantly improving technique, a global approach must be considered, including MRI machines, sequences and products. In other words, with special sequences, such as fast imaging techniques, some compounds can be used as positive or negative enhancers.

Knowledge of the structures/efficacy relationships is absolutely necessary to synthetize the future compounds. For example, the management of the Solomon-Bloembergen equations is very important in order to improve the efficacy of Gadolinium complexes. More efficient products will probably make it possible to reduce the doses and consequently increase the tolerance.

### V. REFERENCES :

(1) : An introduction to MR in Medicine. Ed. P.A. Rinck.Georg. Thieme Verlag Stuttgart NY 1990
(2) : T. Kowall, F. Foglia, L. Helm, A.E. Merbach ; Chem. Eur. J. 1996, 2, n°3, p. 285.
(3) : M.F. Tweedle Invest. Radiology 1992 ; vol. 27 (suppl. 1) : S 2-6.
(4) : M.F. Tweedle Invest Radiology 1995 ; vol. 30. 372

